# Patterns of human exposure to malaria vectors in Zanzibar and implications for malaria elimination efforts

**DOI:** 10.1186/s12936-020-03266-w

**Published:** 2020-06-22

**Authors:** April Monroe, Dickson Msaky, Samson Kiware, Brian B. Tarimo, Sarah Moore, Khamis Haji, Hannah Koenker, Steven Harvey, Marceline Finda, Halfan Ngowo, Kimberly Mihayo, George Greer, Abdullah Ali, Fredros Okumu

**Affiliations:** 1 PMI VectorWorks Project, Johns Hopkins Center for Communication Programs, Baltimore, MD USA; 2 University of Basel, Basel, Switzerland; 3 Swiss Tropical and Public Health Institute, Basel, Switzerland; 4 Environmental Health and Ecological Sciences Department, Ifakara Health Institute, Ifakara, Tanzania; 5 Zanzibar Malaria Elimination Programme, Zanzibar, Tanzania; 6 Department of International Health, Johns Hopkins Bloomberg School of Public Health, Baltimore, MD USA; 7 U.S. President’s Malaria Initiative, U.S. Agency for International Development, Dar Es Salaam, Tanzania; 8 School of Public Health, Faculty of Health Sciences, University of the Witwatersrand, Parktown, Republic of South Africa; 9 Institute of Biodiversity, Animal Health and Comparative Medicine, University of Glasgow, Glasgow, UK

**Keywords:** Malaria, Residual transmission, Outdoor transmission, Human behavior, Zanzibar, Tanzania, Human–vector contact, Human–vector interaction, Exposure

## Abstract

**Background:**

Zanzibar provides a good case study for malaria elimination. The islands have experienced a dramatic reduction in malaria burden since the introduction of effective vector control interventions and case management. Malaria prevalence has now been maintained below 1% for the past decade and the islands can feasibly aim for elimination.

**Methods:**

To better understand factors that may contribute to remaining low-level malaria transmission in Zanzibar, layered human behavioural and entomological research was conducted between December 2016 and December 2017 in 135 randomly selected households across six administrative wards. The study included: (1) household surveys, (2) structured household observations of nighttime activity and sleeping patterns, and (3) paired indoor and outdoor mosquito collections. Entomological and human behavioural data were integrated to provide weighted estimates of exposure to vector bites, accounting for proportions of people indoors or outdoors, and protected by insecticide-treated nets (ITNs) each hour of the night.

**Results:**

Overall, 92% of female *Anopheles* mosquitoes were caught in the rainy season compared to 8% in the dry season and 72% were caught outdoors compared to 28% indoors. For individual ITN users, ITNs prevented an estimated two-thirds (66%) of exposure to vector bites and nearly three quarters (73%) of residual exposure was estimated to occur outdoors. Based on observed levels of ITN use in the study sites, the population-wide mean personal protection provided by ITNs was 42%.

**Discussion/conclusions:**

This study identified gaps in malaria prevention in Zanzibar with results directly applicable for improving ongoing programme activities. While overall biting risk was low, the most notable finding was that current levels of ITN use are estimated to prevent less than half of exposure to malaria vector bites. Variation in ITN use across sites and seasons suggests that additional gains could be made through targeted social and behaviour change interventions. However, even for ITN users, gaps in protection remain, with a majority of exposure to vector bites occurring outdoors before going to sleep. Supplemental interventions targeting outdoor exposure to malaria vectors, and groups that may be at increased risk of exposure to malaria vectors, should be explored.

## Background

Zanzibar provides a good case study for malaria elimination. Despite historically high transmission, the islands experienced a dramatic decline in malaria cases and deaths following the introduction of artemisinin-based combination therapy (ACT) and effective vector control interventions, namely insecticide-treated nets (ITNs) and indoor residual spraying (IRS) [1]. Since 2008, low level transmission has been maintained with malaria prevalence below 1% [1]. Field evidence suggests remaining cases are geographically focused and coincident with areas with high vector abundance [2].

Zanzibar has operationally scaled up core vector control interventions in recent years. Universal coverage campaigns were implemented in 2012 and 2016 with the goal of providing one ITN for every two people. Beginning in 2014, continuous distribution of ITNs through community and health facility-based channels has helped to maintain high levels of access [3]. IRS was introduced in 2007 with the goal of universal coverage, and in 2012, shifted from blanket spraying to targeted deployment in hot spots once a year before the start of the rainy season [1].

In addition to optimizing the impact of core vector control interventions, it is increasingly important to understand factors that can contribute to persistent low-level malaria transmission once high coverage of these interventions has been achieved [4, 5]. Challenges such as increased outdoor biting proportions in response to indoor insecticidal interventions, shifts in peak biting times to early-evening hours before most people are under their ITNs, and human activities outdoors when malaria vectors are active may attenuate the protection provided by ITNs or other indoor interventions [4, 6–9].

To better understand patterns of vector behaviour, entomological monitoring is now carried out in ten sentinel sites in Zanzibar providing valuable data on vector species abundance and distribution as well as insecticide resistance [10]. While entomological monitoring is critical, a more complete understanding of the protection provided by current vector control interventions requires an understanding of how vector behaviour corresponds to human activity and sleeping patterns. This information can provide a clearer picture of when (time of night) and where (indoors or outdoors) people may be exposed to vector bites. As part of a larger study investigating potential drivers of persistent malaria transmission in Zanzibar, this article presents results from layered human behavioural and entomological data collection.

## Methods

### Study area

**Fig. 1:**
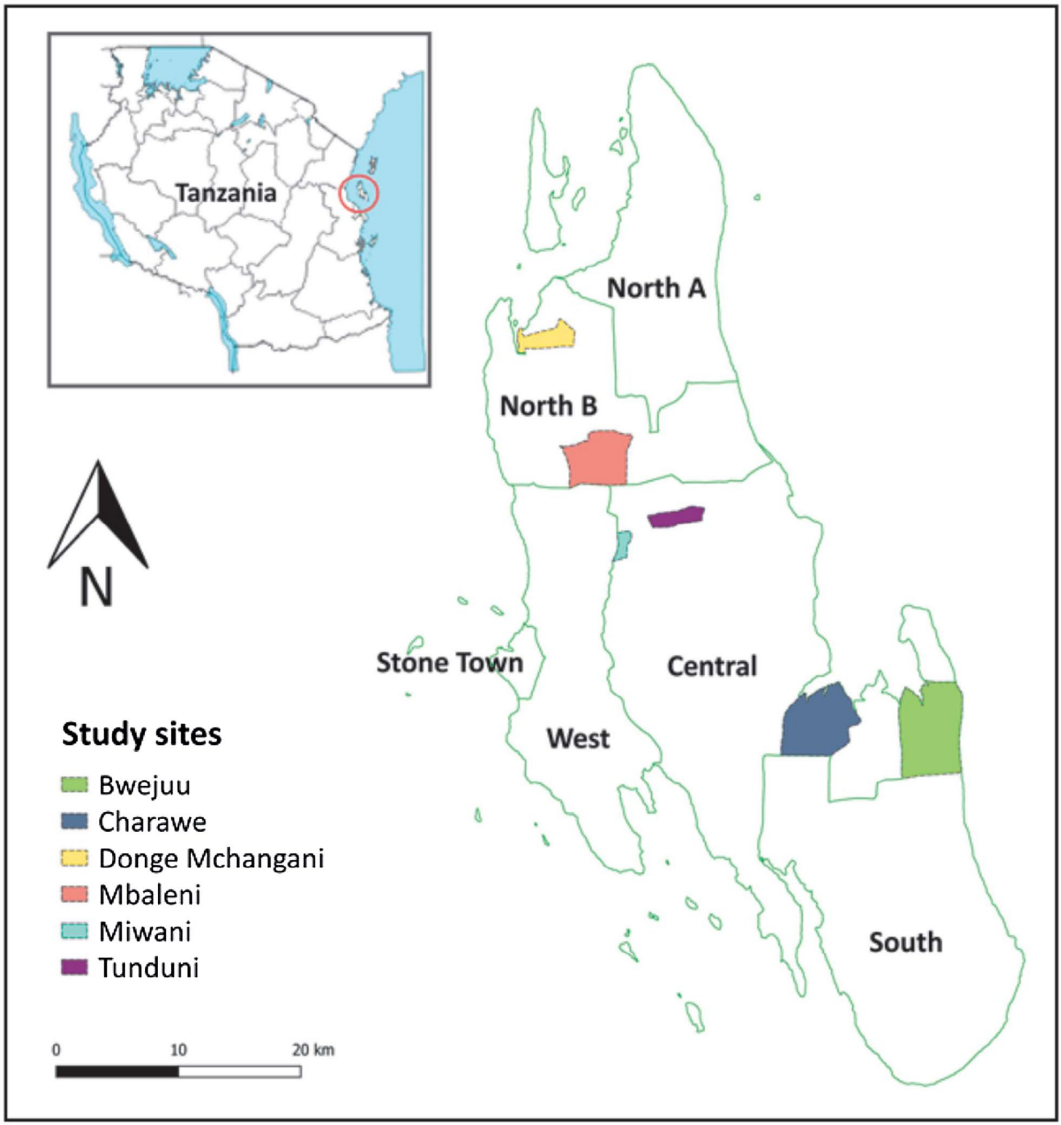
Map of study sites on Unguja Island, Zanzibar

This study took place in six *Shehia* (wards) on Unguja Island, the main island of Zanzibar, an archipelago located off the coast of mainland Tanzania (Fig. 1). Sites were selected in partnership with the Zanzibar Malaria Elimination Programme (ZAMEP) on the basis of high malaria incidence, defined as annual parasite incidence (API) of 5/1000 or higher, and receipt of IRS in 2016.

### Study design

This study included household surveys, structured observations of nighttime human activity and sleeping patterns, and indoor and outdoor mosquito collections. In Zanzibar, there are generally two dry seasons occurring approximately from December through February and June through September. The rainy season, which is characterized by heavy downpours, generally occurs from March through May, with a peak in April. A less pronounced rainy season is also observed from October through November. Human behavioural data were collected in the dry season in December 2016 and rainy season from April through May 2017. Entomological data were collected across 10 months between December 2016 and December 2017. Five months were classified as rainy season (March, April, May, October and November) and the remaining as dry season (January, February, August, September and December). The same households were used for human behavioural and entomological data collection and across data collection time points.

### Sample size

The sample size was generated to answer the primary research question of whether there was a difference in number of malaria vectors biting indoors compared to outdoors each night. The method developed by Cohen for power calculations in behavioural sciences was used [11]. Based on previous estimates from entomological monitoring, an average of 7 *Anopheles arabiensis* caught outdoors and 4 caught indoors was assumed, which translates to medium effect sizes. However, considering potential for heterogeneity in vector biting densities across households, sites, and seasons, effect sizes (measured as R^2^/1 − R^2^) as low as 0.02–0.15 were assumed on a generalized linear model regressing mosquito counts as a function of position (indoors/outdoors as a fixed effect and day and location as random effects to account for heterogeneity in the data). The study was then designed to achieve 80% power at 95% confidence intervals, which returned a requirement for 200 nights of mosquito collection per site.

Based on previous research using direct observation of night time human behaviour, it was determined that 20–25 households per site would be needed to capture variation in human behaviour across households and sites [12]. Therefore, to achieve 200 nights of mosquito collection per site from the same households where human behavioural data was collected, eight nights of indoor and outdoor mosquito collection were carried out in each household. Collection nights were evenly distributed across seasons.

A random number generator was used to select 30 households for each of the six *shehia* (wards) using household listings provided by the *Sheha* (local leader) for each site. Of the 180 households selected, 143 were home, and therefore approached, during community entry. A total of 135 households consented to participate as follows: Bwejuu (n = 20), Charawe (n = 23), Donge Mchangani (n = 24), Mbaleni (n = 23), Miwani (n = 25) and Tunduni (n = 20). Of the households approached, four households declined to participate, two household heads were not available to provide consent, and two indicated that they would be traveling throughout the data collection period.

### Data collection

#### Human behaviour

Study team members administered a survey to respective heads of household prior to beginning night-time observations. The survey included questions on household members, housing characteristics, and bed net ownership. For each person living in the household, information was collected on relationship to head of household, age, sex and pregnancy status, if known. Net ownership and characteristics were recorded using a standard net roster [13].

Study team members made structured observations half-hourly of each individual household member’s activities and sleeping patterns from 6:00 p.m. to 7:00 a.m. This included (a) whether each household member was indoors, outdoors, or away from home, (b) awake or asleep and (c) if sleeping, whether they were using an ITN.

Data from surveys and household observations were recorded electronically using tablets configured with programmed questionnaires and observation forms. These data collection tools were first translated into Swahili and then programmed using the open-source platform, Open Data Kit (ODK) [14]. Data collectors were trained on how to operate tablets, complete the forms, and upload the data. The data was uploaded daily to a secure server configured with Secure Socket Layer (SSL) with encryption. Appropriate logical constraints were implemented on every question to ensure data quality. In addition, for the household observations, time stamps were fixed to block entry of missed observations. Supervisors reviewed data for quality on a daily basis and provided feedback to the data collection team.

#### Vector behaviour

**Fig. 2:**
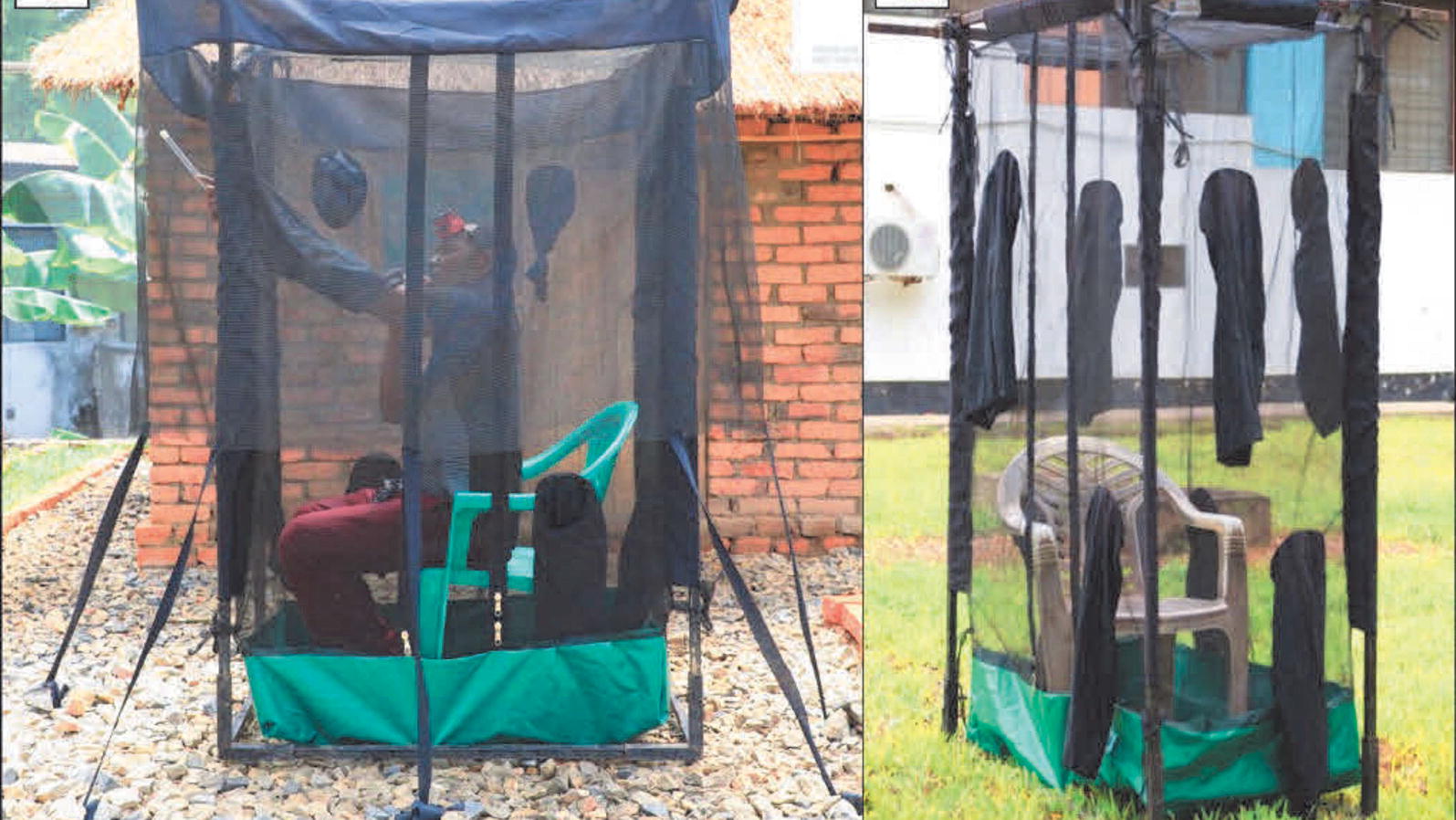
Photo of the miniaturized double net trap used to catch host-seeking mosquitoes indoors and outdoors. The miniaturized double net trap consists of an inner chamber, normally occupied by adult volunteer mosquito catchers. There is an outer netting cover, hanging 80 cm from the ground, which traps host-seeking mosquitoes attempting to reach the volunteer inside. Host-seeking mosquitoes are trapped between the inner and outer netting compartments are collected by the volunteer through the multiple sleeves which open outwards from the inner compartment using a mouth aspirator

Indoor and outdoor mosquito collections were conducted hourly from 6:00 p.m. to 7:00 a.m. in the selected households in each *shehia* using human-baited miniaturized double net traps (DN-mini) (Fig. 2), an exposure-free method developed at Ifakara Health Institute [15]. This trap was developed based on the design previously used by WHO [16] and modified by Tangena et al. [17]. Observations of indoor and outdoor proportions and hourly biting patterns of Anopheles in Tanzania with DN-mini match those of the gold standard estimate of human exposure to mosquito bites the human landing catch [15]. Mosquitoes were collected hourly using a mouth aspirator and put in a paper cup, with a separate cup labeled for each hour of collection. The collectors sampled mosquitoes for 45 min each hour and rested for 15 min. Collectors worked in two sets with each set doing collections indoors and outdoors for 6 to 7 h each night of collection.

Mosquitoes were sorted by taxa, sex, and physiological status (fed, unfed or gravid), and then stored individually or in batches for laboratory analysis. These samples were stored in microcentrifuge tubes containing cotton wool and silica gel, and were later analyzed by Polymerase Chain Reaction (PCR) to distinguish between members of *Anopheles gambiae* sensu lato (s.l.), and by enzyme-linked immunosorbent assays (ELISA) to determine proportions carrying *Plasmodium falciparum* sporozoites in their salivary glands [18]. The field data and laboratory results were recorded electronically using tablets, linked, cleaned, and stored in a secure web-based database application, the Ifakara Entomology Bioinformatics System (IEBS) [19].

### Data analysis

#### Human behaviour

Descriptive analysis of household survey data and observation data was completed using STATA 14 [20] and graphs were generated in Microsoft Excel [21]. ITN access was calculated using the approach originally described by Kilian et al. and recommended by the Roll Back Malaria Monitoring and Evaluation Reference Group [13, 22]. Potential ITN users were calculated by multiplying the number of ITNs in each household by two (assuming a maximum of two users per ITN). If the potential users exceeded the number of people in the household, the number of ITN users was set to the number of household members. ITN access was then calculated by dividing potential ITN users by the total number of study participants [18]. The use to access ratio (UAR) was calculated by dividing the proportion of the study population observed to be using an ITN by the proportion of study population with access to an ITN.

#### Vector behaviour

Mosquito biting patterns were assessed based on hourly catches each night for dry and rainy seasons separately. Collection nights were evenly distributed across seasons. No mosquitoes were infected with *Plasmodium* and, therefore, no calculation was done for the sporozoite rate. The probability of a mosquito biting indoors or outdoors was estimated from a Generalized Linear Mixed Effects Regression (GLMER) with a Poisson distribution with a log link, using household ID and round of collection as random effects and location (in versus out) as a fixed effect. Analysis was done using R statistical package version 3.6.1 [23].

#### Human–vector interaction

Human exposure to malaria vectors was calculated based on data from household observations carried out in the peri-domestic setting and indoor and outdoor mosquito collections in the same households. Exposure patterns were calculated only for *An. gambiae* s.l. as densities of other *Anopheles* complexes were too low to explore patterns of exposure. Analysis included calculation of the following indicators of human–vector interaction, described by Monroe et al. [24].

1. Percentage of vector bites occurring indoors for an unprotected individual ($\pi_{I,u}$) [25–29]
  This is an indicator of the maximum possible protection any indoor intervention could provide. Calculated as the sum of the measured indoor vector biting rates (*B*_*I*_) for each 1-h time period (*t*) over a 24-h period weighted by the estimated proportion of humans indoors (*I*) at that time, divided by total location weighted exposure (indoors and outdoors):
  $$\pi_{I,u} = \frac{{\sum_{t = 1 }^{24} B_{I,t } I_{t} }}{{\sum_{t = 1 }^{24} B_{I,t } I_{t} + B_{O,t} O_{t} }}$$
2. Percentage of vector bites occurring while asleep indoors for an unprotected individual ($\pi_{S,u}$) [25–27, 29, 30]
  An indicator of the maximum possible personal protection an intervention targeting indoor sleeping spaces, such as ITNs, could provide. Calculated as, the sum of the indoor vector biting rates (*B*_*I*_) for each 1-h time period (*t*) over a 24-h period weighted by the estimated proportion of humans sleeping (*S*) indoors at that time, divided by total location weighted exposure:
  $$\pi_{S,u} = \frac{{\sum_{t = 1 }^{24} B_{I,t } S_{t} }}{{\sum_{t = 1 }^{24} B_{I,t } I_{t} + B_{O,t} O_{t} }}$$
3. Percentage of all vector bites directly prevented by using an ITN ($P_{S}^{*}$) [28, 30–33]
  Calculated as the product of the proportion of exposure occurring while asleep and the personal protection against bites (feeding inhibition) provided by an ITN while in use (*ρ).* ITNs were assumed to prevent 97% of vector bites when in use based on reference estimates from experimental hut trials of 7 brands of ITNs in Tanzania [34].
  $$P_{S}^{*} = \rho \pi_{S,u} = \frac{{\rho \sum_{t = 1 }^{24} B_{I,t } S_{t} }}{{\sum_{t = 1 }^{24} B_{I,t } I_{t} + B_{O,t} O_{t} }}$$
4. Percentage of remaining exposure occurring indoors for a protected user of an ITN ($\pi_{I,p}$) [26–29]
  And indicator of where remaining exposure to vector bites occurs for an ITN user (indoors versus outdoors). Calculated by adjusting the estimate of π_I,u_ to allow for the indoor personal protection provided by using an ITN:
  $$\pi_{I,p} = \frac{{(\sum_{t = 1 }^{24} B_{I,t } I_{t} ) - \rho \left( {\sum_{t = 1 }^{24} B_{I,t } S_{t} } \right)}}{{(\sum_{t = 1 }^{24} B_{O,t} O_{t} + B_{I,t } I_{t} ) - \rho \left( {\sum_{t = 1 }^{24} B_{I,t } S_{t} } \right)}}$$
  The percentage of bites occurring outdoors for an ITN user ($\pi_{O,p} = 1 - \pi_{I,p}$) was calculated as:
5. Population-wide mean personal protection against biting exposure provided by observed levels of ITN use (*C*) in the community ($P_{S,C}^{*}$)
  Calculated as the product of the proportion of the population using an ITN at each hour during the night and the overall personal protection provided by an ITN while it is in use, and accounting for the attenuating effects of exposure occurring when the user is active outside the net.
  $$P_{S,C}^{*} = \frac{{\rho \sum_{t = 1 }^{24} B_{I,t } C_{t} }}{{\sum_{t = 1 }^{24} B_{I,t } I_{t} + B_{O,t} O_{t} }} = \rho \pi_{S,p} C$$

### Ethical approval

This study received ethical approval from the Johns Hopkins Bloomberg School of Public Health (IRB# 7390), Ifakara Health Institute (IHI/IRB/No: 035 - 2016), and the Zanzibar Medical Research and Ethics Committee (Protocol #: ZAMREC/0005/OCT/016). Only consenting mosquito collection volunteers participated. Volunteers received appropriate training and were provided with medical supervision, chemoprophylaxis, and access to diagnosis and treatment on a regular basis. Heads of household provided separate written consent for household observations and mosquito collection respectively. Community entry activities were conducted prior to beginning data collection. This included a 1-day information session for *Sheha* (local leaders) and assistant *Sheha* in the selected sites and district-level representatives. During site visits study team members explained the purpose of the study to community members and obtained informed consent from selected heads of household.

## Results

Results are grouped by human behaviour, vector behaviour, and human–vector interaction. Specific result areas include demographic characteristics of household members, nighttime location and sleeping patterns of household members, levels of ITN access and use, indoor and outdoor vector biting patterns and species composition, and finally patterns of human exposure to malaria vectors as a function of human and vector data.

### Demographic characteristics

A total of 699 people was observed across 135 households. The same households were observed once each during the dry and rainy seasons. Participants were roughly evenly split by sex; additional detail on the participant demographic characteristics is provided in Table 1.

**Table 1:** Demographic characteristics of household members

	Male	Female	Total^a^
Household members	331 (47%)	368 (53%)	699
< 1 year	6	10	16 (2%)
1–4 years	36	53	89 (13%)
5–9 years	49	49	98 (14%)
10–17 years	67	65	132 (19%)
18–59 YEARS	156	168	324 (46%)
≥ 60 years	17	23	40 (6%)

^a^Data presented in this table were collected during the first round of data collection in December 2016. A total of 682 of the original 699 household members were observed in the second round of data collection in April–May 2017. Three households did not participate in the rainy season collection; two households were not available, and one household refused

### Nighttime human location and sleeping patterns

#### Time spent away from home

**Fig. 3:**
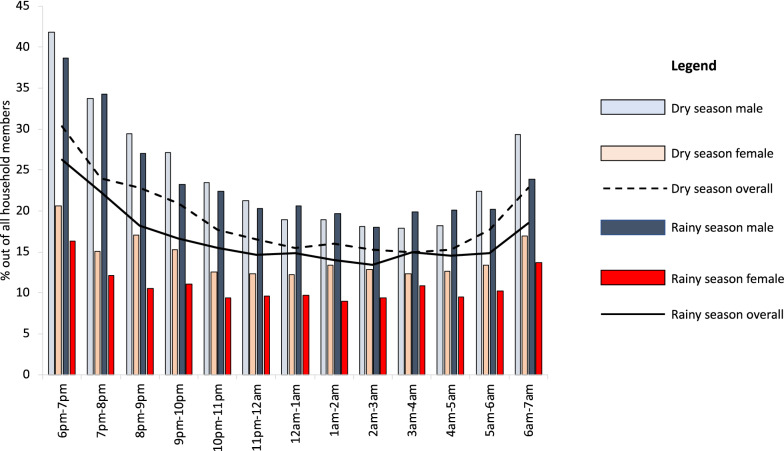
Percentage of males and females away from home throughout the night, across seasons

The percentage of the study population observed as away from home peaked in the early evening with 26–30% away between 6:00 p.m. and 7:00 p.m. and slowly declined. The percentage away was observed to be lowest in the late-night hours, staying steady at approximately 15% from 11:00 p.m. until 4:00 a.m. in both the dry and rainy season before rising again from 4:00 a.m. to 7:00 a.m. Throughout the night, the percentage of males away from home was approximately double that of females, with a peak of 40% of males away in the early evening in dry season and staying constant at approximately 20% in the middle of the night (Fig. 3).

#### Time spent in the peri-domestic space

**Fig. 4:**
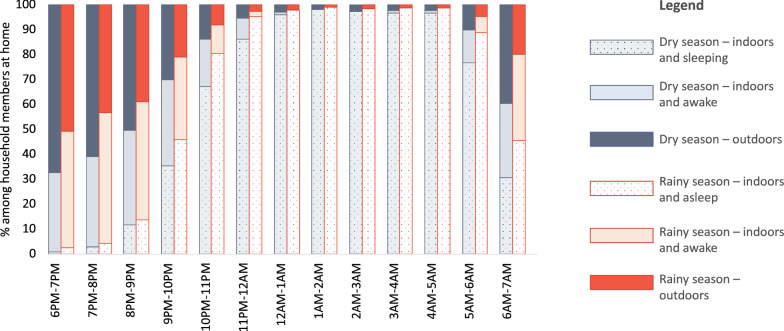
Percentage of people outdoors, indoors and awake, and indoors and sleeping throughout the night across seasons, among household members who were observed within the peri-domestic space

Among study participants observed indoors and directly outside of the home, the percentage of the population outdoors peaked in the early evening hours, with 67% outdoors in the dry season and 51% outdoors in the rainy season between 6:00 p.m. and 7:00 p.m. The percentage of the population outdoors slowly declined and stayed steady at less than 5% between 11:00 p.m and 4:00 a.m., when nearly all household members at home were recorded to be indoors and asleep, before beginning to rise again in the early morning between 4:00 a.m. and 7:00 a.m. (Fig. 4).

#### ITN access and use

Assuming one ITN can be used by two people, approximately three quarters (76%) of the study population had access to an ITN in their household. ITN access varied by site, with the lowest level recorded in Miwani and the highest recorded in Bwejuu (Table 2).

**Table 2:** Mean ITN access, use, and use:access ratio (UAR) during peak sleeping hours (11:00 pm–4:00am) across season and *shehia*

Shehia	Dry season			Rainy season		
	ITN access (%)	ITN use (%)	UAR (%)	ITN access (%)	ITN use (%)	UAR (%)
Bwejuu	94	79	84	93	68	73
Charawe	73	76	104	68	78	115
Donge Mchangani	72	54	76	79	67	85
Mbaleni	74	57	77	71	80	112
Miwani	69	2	42	63	45	71
Tunduni	79	44	56	79	28	36
Total	76	56	74	75	62	82

**Fig. 5:**
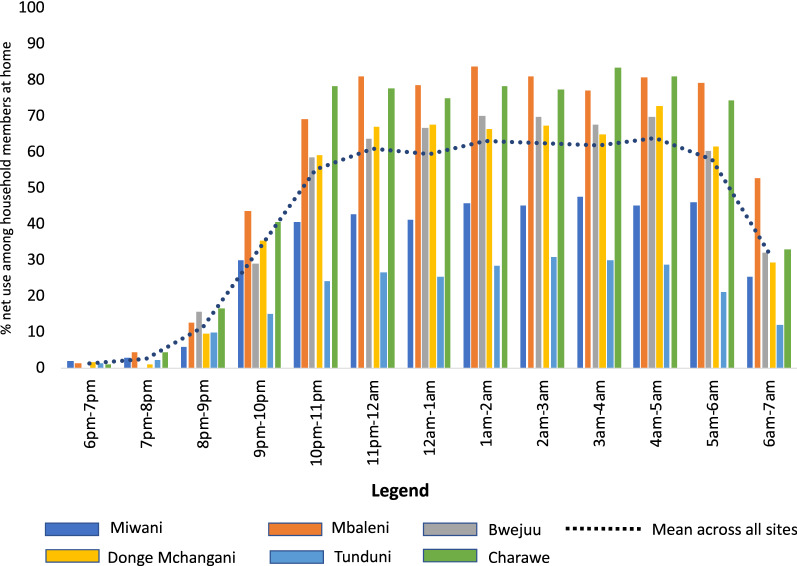
Average percentage of ITN use by hour across *shehia* observed in the rainy season, among participants in the peri-domestic space

**Fig. 6:**
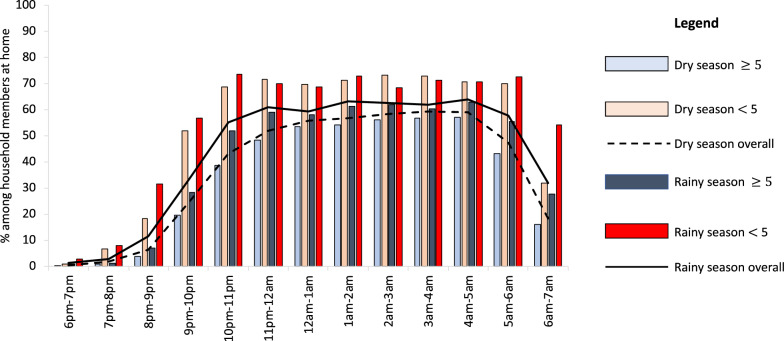
Average level of ITN use for participants aged under 5 years and 5 years and over, by hour, across seasons

Among household members at home, ITN use was highest during peak sleeping hours, between 11:00 p.m. and 4:00 a.m. Average ITN use during this time was 56% in the dry season and 62% in the rainy season. Variability was observed across study sites with the lowest levels of net use observed in Miwani and Tunduni across dry and rainy season with an average level of net use of 29% recorded in Miwani in the dry season and 28% in Tunduni in the rainy season during peak sleeping hours. The highest average net use was observed in Bwejuu (79%) and Charawe (76%) in the dry season and Mbaleni (80%) and Charawe (78%) in the rainy season (Fig. 5). On average, a higher percentage of household members under 5 years used an ITN with over 70% net use during peak sleeping hours in both the dry and rainy seasons, compared to participants aged 5 years and older who had an average ITN use of 54% in the dry season and 61% in the rainy season (Fig. 6). The UAR during peak sleeping hours was 74% in the dry season and 82% in the rainy season, with lowest levels recorded in Miwani and Tunduni (Table 2).

### Malaria vector species diversity and biting patterns

A total of 343 female *Anopheles* were collected using the miniaturized double net trap; 92% of all *Anopheles* were collected in the rainy season. Of the *Anopheles* caught with the double net trap, the mean vector biting was the highest in Mbaleni, followed by Miwani and Donge Mchangani. No *Anopheles* were caught using this method in the other three sites. Of all *Anopheles*, 72% (n = 248) were caught outdoors, while 28% (n = 95) were caught indoors. Within the three sites where *Anopheles* were caught, the number of mosquitoes caught was 56% higher outdoors than indoors in Mbaleni, 85% higher in Donge Mchangani, and more than twice as high in Miwani (Table 3).

**Table 3:** Rate ratio and 95% confidence interval for mosquitoes caught indoors and outdoors, by *shehia*

Shehia	Location	No. of female *Anopheles*	Rate ratio [95% CI]	P-values
Mbaleni	Indoor	62	1	
	Outdoor	137	1.56 [1.13, 2.14]	< 0.01*
Donge Mchangani	Indoor	9	1	
	Outdoor	21	1.85 [0.83, 4.11]	0.129
Miwani	Indoor	24	1	
	Outdoor	90	2.33 [1.37, 3.98]	< 0.01*

*Significant difference at the 0.01 level

*Anopheles gambiae* s.l. was the most common vector species, accounting for 84% (n = 289) of malaria vectors caught using this method. PCR analysis was carried out for 284 of the *An. gambiae* s.l. samples, of which over 98% were identified as *An. arabiensis* (n = 280) and the remaining were *Anopheles merus* (n = 2) and *An. gambiae* sensu stricto (n = 2). Other *Anopheles* species caught included *Anopheles squamosus* (5 females indoors and 48 females outdoors) and *Anopheles coustani* (1 female outdoors and none indoors). No *Plasmodium* sporozoite positive mosquitoes were identified.

### Patterns of human–vector interaction inside and directly outside of the home

**Fig. 7:**
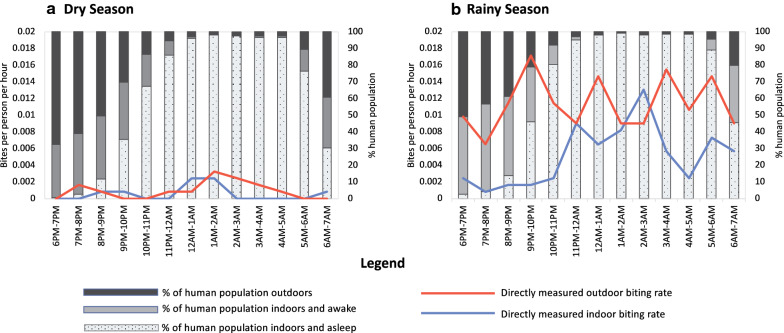
Proportion of human population indoors and awake, indoors and asleep, and outdoors throughout the night, overlaid with directly measured indoor and outdoor biting rates for *Anopheles gambiae* s.l. across seasons. Of *An. gambiae* s.l. over 98% were *Anopheles arabiensis*

Outdoor biting rates remained relatively consistent throughout the night, while indoor biting peaked in the middle of the night when the highest percentage of the human population was observed to be indoors (Fig. 7).

**Fig. 8:**
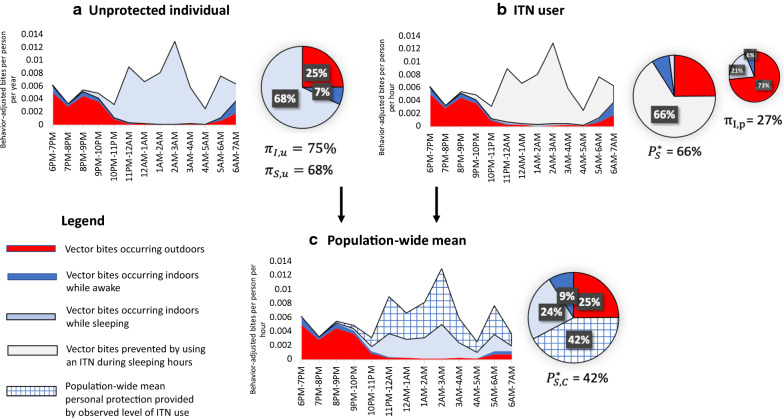
Average pattern of exposure to *Anopheles gambiae* s.l. bites throughout the night in the rainy season for **a** unprotected individuals, **b** individuals who use an ITN while asleep, and **c** the population-wide mean exposure to vector bites based on the observed level of ITN use in the study population throughout the night. Of *An. gambiae* s.l., over 98% were *Anopheles arabiensis*

For an unprotected individual, defined as a person who did not use an ITN at any time during the night, an estimated 79% and 75% of exposure to vector bites occurred indoors ($\pi_{I,u}$) in the dry and rainy seasons, respectively, and 68% occurred while indoors and asleep ($\pi_{S,u}$) across seasons (Table 4), with indoor exposure peaking in the middle of the night (Fig. 8).

**Table 4:** Human exposure patterns to *Anopheles gambiae s.l.* bites by season

Indicator	Dry season	Rainy season
Exposure for an unprotected individual		
Percentage of vector bites occurring indoors for an unprotected individual ($\pi_{I,u}$)	79%	75%
Percentage of vector bites occurring while asleep indoors for an unprotected individual ($\pi_{S,u}$)	68%	68%
Exposure prevented by ITN use		
Percentage of all vector bites prevented by using an ITN ($P_{S}^{*}$)	66%	66%
Remaining exposure for an ITN-user		
Percentage of remaining exposure occurring indoors ($\pi_{l,p}$) and (outdoors) ($\pi_{O,p}$) for a protected user of an ITN	39% (61%)	27% (73%)
Population mean exposure based on observed level of net use		
Population-wide mean personal protection against biting exposure provided by observed level of ITN use $P_{{S,C_{h} }}^{*}$	39%	42%

Use of an ITN while asleep was estimated to directly prevent 66% of exposure to malaria vector bites out of all exposure that would otherwise occur ($P_{S}^{*}$) and for an ITN user, a majority of remaining exposure was estimated to occur outdoors ($\pi_{O,p}$) (Table 4) in the evening hours before sleeping (Fig. 8). When accounting for the percentage of the study population using an ITN for every hour of the night (Fig. 5), the population mean personal protection provided by observed levels of ITN use was 39% and 42% in the dry and rainy season respectively (Table 4 and Fig. 8).

## Discussion

A better understanding of intervention use, human activity and sleeping patterns, and how they overlap with local vector behaviour, can provide an improved understanding of persistent malaria transmission and guide interventions to protect people when and where they need it. While increasing and sustaining ITN access and use is critical across settings, malaria control and elimination programmes should also consider the limitations of current interventions.

Perhaps the most important finding from this work was that current levels of ITN use are estimated to directly prevent less than half of exposure to malaria vector bites. Remaining exposure to vector bites is likely driven by both sub-optimal levels of ITN use in some sites as well as exposure that cannot be prevented by ITN use. Average levels of ITN access and UAR were above 70% across seasons, which is relatively high compared to other settings in sub-Saharan Africa [35, 36]. However, variation in levels of use was observed across locations suggesting additional gains could be achieved in some communities. Distribution and promotion of ITNs should continue across sites, with targeted social and behaviour change interventions focused on locations with lower access and UAR such as Miwani and Tunduni.

In addition to optimizing the impact of core vector control interventions, it is important to consider gaps that remain. For ITN users, approximately three quarters of remaining exposure occurred outdoors, largely in the hours before sleeping. Qualitative research findings from in-depth interviews and direct observation of nighttime community events in the same study sites provide in-depth information on nighttime activities that can help to inform context-appropriate interventions [37]. Common nighttime activities in these sites included small-scale routine social activities such as gathering to socialize and play cards in the evening, watching television and football matches next to small shops, and entertainment such as going to bars on the weekend. Livelihood activities, lasting all or part of the night, were also commonly reported including security jobs, hunting, and working in hotels or fishing in coastal areas, as well as staying outdoors to guard crops from theft before harvest. Large-scale events, such as weddings, funerals, and religious events, were observed and reported to last all or most of the night [37].

Other studies have found challenges to malaria prevention away from home, including logistic and social barriers to ITN use [12, 38]. However, the feasibility of intervention use may differ depending on the nature of activity. For example, ITN use could be promoted while traveling or visiting friends and family, while supplemental prevention measures would likely be needed to protect people during activities such as socio-cultural events, nighttime occupations, and entertainment which often occur outdoors.

Although the World Health Organization (WHO) does not yet recommend the large-scale deployment of supplemental vector control tools, research is underway to evaluate the effectiveness of interventions such as topical and spatial repellents, insecticide-treated clothing, and improved housing, as well as attractive targeted sugar baits, outdoor traps, and systemic insecticides applied to livestock [39]. Larval source management, which could reduce both indoor and outdoor-biting vector populations, is another option that could be considered. Operational research could be useful in Zanzibar and beyond to better understand where and how to deploy these supplemental tools for maximum impact.

An increasing number of countries are now within reach of malaria elimination with 46 countries reporting fewer than 10,000 indigenous cases in 2017 [36]. The epidemiology of malaria has changed in many of these contexts, with cases increasingly clustered geographically and among certain demographic groups [40]. Often, a high proportion of cases are observed among men and hard to reach groups, such as migrant populations, and current malaria interventions are unlikely to adequately address these changes [40]. In Zanzibar, a higher percentage of males was recorded to be away throughout the night compared to females, and qualitative research findings suggest males are more likely to engage in nighttime occupations, to travel, and to stay outdoors later socializing at night, all of which may impact exposure to malaria vectors. Likewise, travel to and from mainland Tanzania has been found to be a risk factor for malaria infection in Zanzibar [1].

In these contexts, effective targeting of interventions is critical and finer scale information on the epidemiological, ecological, and socio-cultural context is needed, including identification of locations and groups at risk [41]. Additional investigation to better understand networks of higher-risk groups and scenarios, research to link specific activities to malaria infection, and programmes targeting these groups with appropriate packages of interventions could be explored in low transmission settings such as Zanzibar.

This study builds on previous studies that have quantified human–vector interaction [25, 27, 29, 32] to provide programmatically useful information on when and where people are exposed to malaria vectors as well as the activities that may put people at risk. Sites were selected on the basis of having high API in the context of high coverage of ITNs and IRS. However, variation was observed in both vector and human behaviour across sites. This finding suggests the value of vector and human behavioural data at the community level to inform targeting of interventions to address specific gaps in protection, particularly in low transmission settings.

Despite the importance of human behaviour to understanding patterns of risk, a review of published literature on nighttime human behaviour found fewer than a dozen studies over the past two decades that integrated human and vector data [42]. Collecting human and vector data together can provide an improved understanding of exposure patterns and inform when and where supplemental tools might be needed and could be considered in future entomological monitoring and research activities.

### Limitations

This work has a number of limitations. Recruitment of households took place on 1 day in each site. Households that were away during the time of recruitment or that would be traveling when data collection began were not included in the study. It is possible that the households that were present to consent on the day of recruitment were different from the households that were not or that households that consented may have been different from the few households than those that refused. However, the study team worked with community leaders to schedule recruitment activities during times when a majority of households were likely to be home.

Further, the recorded biting rates may have been impacted by the trapping method used. While, a study by Tangena et al. found no significant difference between numbers of *Anopheles* mosquitoes caught by double net trap and human landing catch [17], the version used in this study had some design differences including its size. When tested by Ifakara Health Institute, the absolute numbers of mosquitoes collected were much lower for the miniaturized double net trap compared to HLC, however indoor and outdoor biting proportions, hourly biting patterns, and species diversities matched previous indoor and outdoor estimates obtained using HLC from the same villages [15]. Despite the potential limitation on absolute numbers, the miniaturized double net trap provided the benefit of an exposure-free option for mosquito collectors, increasing the safety of their work while still allowing the relative biting risk indoors and outdoors to be estimated.

Another potential limitation is where mosquitoes were collected. Mosquito collections were carried out in the peri-domestic setting, leaving a gap in data for places people go when away from home, within their community and beyond. Likewise, it was not possible to measure time spent outdoors or under an ITN for people who were recorded to be away from home. Given that many nighttime activities away from home occur outdoors, the estimate of human exposure to malaria vectors occurring indoors and prevented by ITN use in the peri-domestic setting is likely an over-estimate for the study population as a whole. This finding underscores the importance of addressing outdoor exposure in this context, both in the peri-domestic setting and away from home, and the potential value of mosquito collections in places where people frequently gather at night.

When utilizing direct observation, there is also the potential for reactivity, a phenomenon in which people change their behaviour due to the presence of an observer [43]. However, reactivity tends to decrease with the length of the observation and in previous studies was found to have little impact on behaviours of interest [44, 45].

Finally, this study did not look at parasite prevalence in the human population or link exposure to vector bites to malaria infection. There is an opportunity to do so in the future for a more complete picture of residual malaria transmission dynamics in Zanzibar and beyond. Despite the limitations, this study provided a high level of information on human behaviour as it relates to exposure to malaria vectors.

## Conclusions

In contexts such as Zanzibar, where malaria elimination is in sight, it becomes increasingly important to target interventions effectively. Understanding human behaviour and where it intersects with vector behaviour will be important for getting to zero locally acquired cases. In the study sites, overall access to ITNs was high and estimated exposure to malaria vectors was low. Opportunities were identified in specific locations and among certain groups to optimize access to and use of ITNs. Additional gaps in protection were identified when participants were outdoors and away from home. The proportion of exposure to malaria vectors occurring outside of sleeping hours suggests that testing of supplemental tools could be explored to enhance elimination efforts. These results should be taken together with data on travel and migration patterns as well as malaria infection dynamics to guide context-appropriate malaria interventions.

## Data Availability

The datasets used and/or analyzed during the current study are available from the corresponding author on reasonable request.

## Abbreviations

ACT: Artemisinin-based combination therapy
API: Annual parasite incidence
ELISA: Enzyme-linked immunosorbent assays
IEBS: Ifakara entomology bioinformatics system
IRS: Indoor residual spraying
ITN: Insecticide-treated mosquito net
PCR: Polymerase chain reaction
UAR: Use:access ratio
ZAMEP: Zanzibar malaria elimination programme

## References

[1] Björkman A, Shakely D, Ali A, Morris U, Mkali H, Abbas A (2019). From high to low malaria transmission in Zanzibar—challenges and opportunities to achieve elimination. BMC Med..

[2] Hardy A, Mageni Z, Dongus S, Killeen G, Macklin MG, Majambare S (2015). Mapping hotspots of malaria transmission from pre-existing hydrology, geology and geomorphology data in the pre-elimination context of Zanzibar, United Republic of Tanzania. Parasit Vectors..

[3] PMI. Tanzania Malaria Operational Plan 2019. U.S. President’s Malaria Initiative; 2019. https://www.pmi.gov/docs/default-source/default-document-library/malaria-operational-plans/fy19/fy-2019-tanzania-malaria-operational-plan.pdf?sfvrsn=3. Accessed 15 Jan 2019.

[4] Durnez L, Coosemans M. Residual transmission of malaria: an old issue for new approaches. 2013. In: Manguin S, editor. Anopheles mosquitoes–new insights into malaria vectors, Chapt 21, 2013. p. 671–704.

[5] Killeen GF (2014). Characterizing, controlling and eliminating residual malaria transmission. Malar J..

[6] Govella NJ, Ferguson H (2012). Why use of interventions targeting outdoor biting mosquitoes will be necessary to achieve malaria elimination. Front Physiol..

[7] Matowo NS, Munhenga G, Tanner M, Coetzee M, Feringa WF, Ngowo HS (2017). Fine-scale spatial and temporal heterogeneities in insecticide resistance profiles of the malaria vector, Anopheles arabiensis in rural south-eastern Tanzania. Wellcome Open Res..

[8] Reddy MR, Overgaard HJ, Abaga S, Reddy VP, Caccone A, Kiszewski AE (2011). Outdoor host seeking behaviour of *Anopheles gambiae* mosquitoes following initiation of malaria vector control on Bioko Island, Equatorial Guinea. Malar J..

[9] Russell TL, Govella NJ, Azizi S, Drakeley CJ, Kachur SP, Killeen GF (2011). Increased proportions of outdoor feeding among residual malaria vector populations following increased use of insecticide-treated nets in rural Tanzania. Malar J..

[10] PMI. Tanzania Malaria Operational Plan FY 2018. President’s Malaria Initiative Tanzania; 2018. https://www.pmi.gov/docs/default-source/default-document-library/malaria-operational-plans/fy-2018/fy-2018-tanzania-malaria-operational-plan. Accessed 15 Jan 2019.

[11] Cohen J. Statistical power analysis for the behavioral sciences. Routledge; 2013.

[12] Monroe A, Asamoah O, Lam Y, Koenker H, Psychas P, Lynch M (2015). Outdoor-sleeping and other night-time activities in northern Ghana: implications for residual transmission and malaria prevention. Malar J..

[13] Household survey indicators for malaria control. MEASURE Evaluation, MEASURE DHS, President’s Malaria Initiative, Roll Back Malaria Partnership, UNICEF, World Health Organization. 2013. https://www.measureevaluation.org/resources/publications/ms-13-78. Accessed 15 Jan 2019.

[14] Hartung C, Lerer A, Anokwa Y, Tseng C, Brunette W, Borriello G. Open data kit: tools to build information services for developing regions. In: Proceedings of the 4th ACM/IEEE international conference on information and communication technologies and development. 2010;18.

[15] Limwagu AJ, Kaindoa EW, Ngowo HS, Hape E, Finda M, Mkandawile G (2019). Using a miniaturized double-net trap (DN-Mini) to assess relationships between indoor–outdoor biting preferences and physiological ages of two malaria vectors, *Anopheles arabiensis* and *Anopheles funestus*. Malar J..

[16] WHO (1975). Manual on practical entomology in Malaria. Part II. Methods and techniques.

[17] Tangena J-AA, Thammavong P, Hiscox A, Lindsay SW, Brey PT (2015). The human-baited double net trap: an alternative to human landing catches for collecting outdoor biting mosquitoes in Lao PDR. PLoS ONE..

[18] Beier JC, Copeland RS, Onyango FK, Asiago CM, Ramadhan M, Koech DK (1991). *Plasmodium* species identification by ELISA for sporozoites removed from dried dissection slides. J Med Entomol.

[19] Kiware SS, Russell TL, Mtema ZJ, Chaki P, Lwetoijera D, Chanda J (2016). A generic schema and data collection forms applicable to diverse entomological studies of mosquitoes. Source Code Biol Med.

[20] Stata: Stata Statistical Software: Release 14. StataCorp; 2015.

[21] Excel: Microsoft Excel Version 16.18. 2018.

[22] Kilian A, Koenker H, Paintain L (2013). Estimating population access to insecticide-treated nets from administrative data: correction factor is needed. Malar J..

[23] R: a language and environment for statistical computing. Vienna: R Foundation for statistical computing; 2019.

[24] Monroe A, Moore S, Okumu F (2020). Methods and indicators for measuring patterns of human exposure to malaria vectors. J Malar.

[25] Bayoh MN, Walker ED, Kosgei J, Ombok M, Olang GB, Githeko AK (2014). Persistently high estimates of late night, indoor exposure to malaria vectors despite high coverage of insecticide treated nets. Parasit Vectors..

[26] Huho B, Briët O, Seyoum A, Sikaala C, Bayoh N, Gimnig J (2013). Consistently high estimates for the proportion of human exposure to malaria vector populations occurring indoors in rural Africa. Int J Epidemiol.

[27] Killeen GF, Kihonda J, Lyimo E, Oketch FR, Kotas ME, Mathenge E (2006). Quantifying behavioural interactions between humans and mosquitoes: evaluating the protective efficacy of insecticidal nets against malaria transmission in rural Tanzania. BMC Infect Dis.

[28] Moiroux N, Damien GB, Egrot M, Djenontin A, Chandre F, Corbel V (2014). Human exposure to early morning *Anopheles funestus* biting behavior and personal protection provided by long-lasting insecticidal nets. PLoS ONE.

[29] Seyoum A, Sikaala CH, Chanda J, Chinula D, Ntamatungiro AJ, Hawela M (2012). Human exposure to *Anopheline* mosquitoes occurs primarily indoors, even for users of insecticide-treated nets in Luangwa Valley, South-east Zambia. Parasit Vectors..

[30] Kamau A, Mwangangi JM, Rono MK, Mogeni P, Omedo I, Midega J (2018). Variation in the effectiveness of insecticide treated nets against malaria and outdoor biting by vectors in Kilifi, Kenya. Wellcome Open Research..

[31] Cooke MK, Kahindi SC, Oriango RM, Owaga C, Ayoma E, Mabuka D (2015). ‘A bite before bed’: exposure to malaria vectors outside the times of net use in the highlands of western Kenya. Malar J..

[32] Geissbühler Y, Chaki P, Emidi B, Govella NJ, Shirima R, Mayagaya V (2007). Interdependence of domestic malaria prevention measures and mosquito–human interactions in urban Dar es Salaam, Tanzania. Malar J..

[33] Thomsen EK, Koimbu G, Pulford J, Jamea-Maiasa S, Ura Y, Keven JB (2016). Mosquito behavior change after distribution of bednets results in decreased protection against malaria exposure. J Infect Dis.

[34] Lorenz LM, Bradley J, Yukich J, Massue DJ, Mboma ZM, Pigeon O, et al. Comparative functional survival and equivalent annual cost of three long lasting insecticidal net (LLIN) products in Tanzania: a three-year prospective cohort study of llin attrition, physical integrity, and insecticidal activity. medRxiv Preprint, 2019. 10.1101/19002212.

[35] Koenker H, Ricotta E, Olapeju B, Choiriyyah I. ITN Access and Use Report - 2018. Baltimore, MD, PMI, VectorWorks Project, Johns Hopkins Center for Communication Programs; 2018.

[36] WHO (2018). World malaria report 2018.

[37] Monroe A, Mihayo K, Okumu F, Finda M, Moore S, Koenker H (2019). Human behaviour and residual malaria transmission in Zanzibar: findings from in-depth interviews and direct observation of community events. Malar J..

[38] Monroe A, Harvey SA, Lam Y, Muhangi D, Loll D, Kabali AT (2014). “People will say that I am proud”: a qualitative study of barriers to bed net use away from home in four Ugandan districts. Malar J..

[39] WHO (2019). Guidelines for malaria vector control.

[40] Cotter C, Sturrock HJ, Hsiang MS, Liu J, Phillips AA, Hwang J (2013). The changing epidemiology of malaria elimination: new strategies for new challenges. Lancet.

[41] WHO (2017). A framework for malaria elimination.

[42] Monroe A, Moore S, Koenker H, Lynch M, Ricotta E (2019). Measuring and characterizing night time human behaviour as it relates to residual malaria transmission in sub-Saharan Africa: a review of the published literature. Malar J..

[43] Bernard HR (2012). Social research methods: Qualitative and quantitative approaches.

[44] Gittelsohn J, Shankar AV, West KP, Ram RM, Gnywali T (1997). Estimating reactivity in direct observation studies of health behaviors. Human Organization..

[45] Harvey SA, Olórtegui MP, Leontsini E, Winch PJ (2009). “They’ll change what they’re doing if they know that you’re watching”: measuring reactivity in health behavior because of an observer’s presence—a case from the Peruvian Amazon. Field Methods..

